# Supplementary figures and images for: The sex lives of ctenophores: the influence of light, body size, and self-fertilization on the reproductive output of the sea walnut, Mnemiopsis leidyi
Source: PeerJ. 2016 Mar 24;4:e1846. doi: 10.7717/peerj.1846 (PMC4811168; doi:10.7717/peerj.1846)

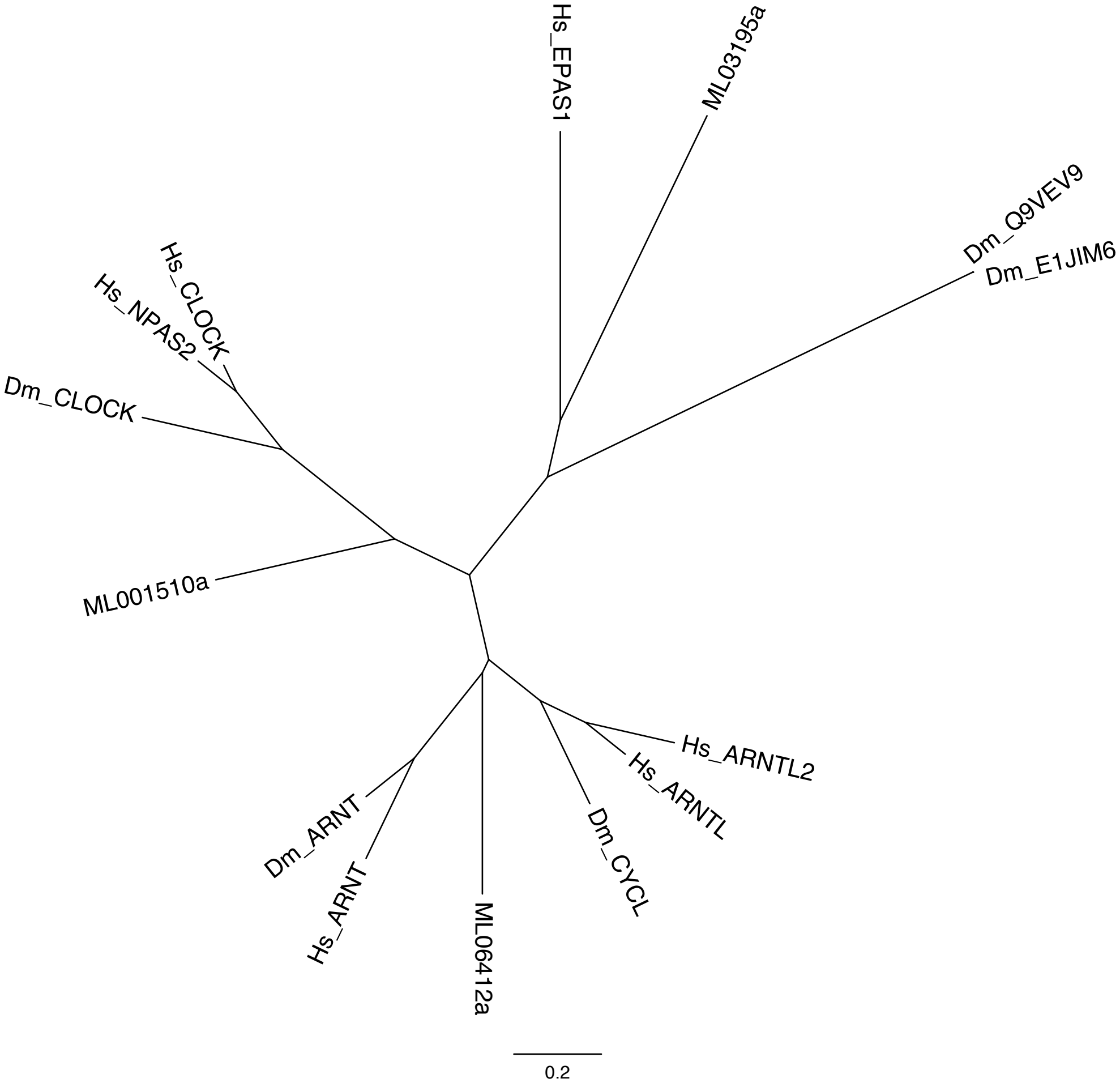

Supplement: Supplemental Information 2 — Genes were aligned with MAFFT version 7 (default settings online); alignments were trimmed with GBlocks version 0.91b (all less stringent settings checked in online version); Maximum likelihood tree was generated with RAxML version 8.1.21 with the following command (raxmlHPC -p 12345 -m PROTGAMMAAUTO -s aln.phy -n AUTO). Genes used are as follows: Hs_NPAS2 (XP_005264011.1),Hs_ARNTL (XP_006718296.1),Hs_ARNTL2 (XP_011519068.1),Hs_EPAS1 (NP_001421.2), Hs_ARNT (NP_001184254.1),Dm_CLOCK (CLOCK_DROME),Dm_ANRT (ARNT_DROME),Dm_CYCL (CYCL_DROME),Dm_Q9VEV9 (Q9VEV9_DROME),Dm_E1JIM6 (E1JIM6_DROME). [file peerj-04-1846-s002.pdf]
